# The severity and associated factors of participation restriction among community-dwelling frail older people: an application of the International Classification of Functioning, Disability and Health (WHO-ICF)

**DOI:** 10.1186/s12877-017-0422-7

**Published:** 2017-01-31

**Authors:** Justina Yat Wa LIU

**Affiliations:** Centre for Gerontological Nursing, School of Nursing, The Hong Kong Polytechnic University, Hung Hom, Hong Kong

**Keywords:** WHO-ICF, Frailty, Participation restriction, Community-dwelling older people

## Abstract

**Background:**

The International Classification of Functioning, Disability, and Health (WHO-ICF) describes participation restriction as one aspect of disability. Participation restriction refers to health problems that can hinder people’s involvement in different life events. It is rational to believe that the prevalence of participation restriction increases among a frail population. However, information about the level of participation restriction among older people, particularly the pre-frail or frail, remains scant. The aim of this study was to identify the prevalence and underlying risk factors associated with participation restriction among community-dwelling frail and pre-frail older people.

**Methods:**

A cross-section of 299 community-dwelling frail older people with a mean age of 79.5 participated in this study. They had to have been identified as being either pre-frail or frail based on the five common characteristics of the frailty phenotype. Their level of participation restriction was assessed based on the Chinese Reintegration to Nursing Living Index (C-RNLI). All other independent variables were identified and systematically linked to different components in the WHO-ICF framework.

**Results:**

Among all participants, 207 (69.2%) were identified as encountering participation restrictions in at least one aspect of their life, with a mean C-RNLI score of 68.3 (SD 19.43). A multivariate regression analysis showed that the participants’ status of frailty, self-perceived social status, level of exhibited depressive mood, sleep quality, mobility, level of fear of falling, and physical activity levels had a significant association with participation restriction. When all of the variables, regardless of significance, were included, the factors together explained 67.1% of the variance in the participants’ participation restriction.

**Conclusion:**

Participation restriction was prevalent among community-dwelling frail older people and was associated with factors across different components in the WHO-ICF. This finding supports the view that participation restriction is multifactorial in nature.

## Background

The aim behind the International Classification of Functioning, Disability, and Health (WHO-ICF) is to provide a standard language and conceptual basis for defining, exploring, and assessing human physio-psycho-social functioning in relation to disability [1]. Under the WHO-ICF framework, disability is a condition with multiple dimensions that develops as a process with the potential to impair body functions and structures (including both the physiological system or anatomical structures), limit daily activities (i.e., encountering difficulties when attempting to perform individual tasks or actions), and restrict community participation (i.e., experiencing problems during involvement in life situations) [2, 3]. All of these aspects of disability interact dynamically with the health of individuals, and with their personal and environmental factors [1]. Among these three aspects of disability, a person’s level of participation restriction is seldom viewed as an indicator of the condition of that person’s health. Thus, it is seldom assessed or explored in either clinical or research settings, particularly where the person under assessment is an older person.

Only a few studies exploring the risk factors of participation restriction among older people in general reported that being older, exhibiting more depressive moods, poor mobility, and a lack of balance confidence were significantly associated with participation restriction [4–6]. It is rational to believe that the prevalence of participation restriction is greater among a frail population. Frailty refers to a physiological state of increased vulnerability to stressors resulting from a decrease and possible dysregulation of reserves in multiple physiological and/or biological systems [7, 8]. The early stages of frailty may be clinically silent, with 32.3% of frail older people having neither disabilities nor comorbidities and maintaining a certain level of independence [8].

However, only one study targeted this specific vulnerable group of older people, and reported that about 80% of community-dwelling frail older people had some form of participation restriction in their life [9]. A multivariate regression analysis generated in Fairhall et al.’s study [9] showed that grip strength, mood, number of medical conditions, and mobility were significantly associated with participation. However, their multivariate model could only explain 29% of the variance in participation restriction. That indicates that participation restriction in frail older people is complicated and can be influenced by different factors related to bio-physio-psychosocial factors. Therefore, more studies are still necessary to comprehensively explain participation restriction among the frail population. Information related to the severity and features of participation restriction may pave the way to developing interventions to address problems related to participation restriction in frail older people. In view of this, the aim in this cross-sectional study was to identify the prevalence and underlying risk factors associated with participation restriction among community-dwelling frail and pre-frail older people.

## Methods

### Population and procedures

Two hundred and ninety-nine participants were recruited from 14 district community and day care health centers between June 2015 and January 2016. A convenience and snowball sampling method was used to recruit community-dwelling older people aged 70 or above and able to communicate in Cantonese. They had to meet the five criteria for pre-frailty or frailty based on the frailty phonotype [10], including i) unintentional weight loss: a self-reported unintentional loss of 10% of body weight in the past year; ii) exhaustion: by answering “Yes” to either “I felt that everything I did was an effort” or “I could not get going in the last week”; iii) slowness: a 4.5-meter walk with an average walking speed in the lowest quintile stratified by median body height; iv) weakness: with a maximal grip strength, as measured by hand dynamometers, in the lowest quintile stratified by the body mass index quartile; and v) low activity: a Physical Activity Scale for the Elderly - Chinese (PASE-C) score in the lowest quintile (i.e., < 30 for men and < 27.5 for women). The presence of 1 - 2 items is an indication of pre-frailty and ≥ 3 items is an indication of frailty [7]. Older people were excluded if they did not speak Cantonese, had cognitive impairment with a score of 6 or below in the Abbreviated Mental Test (AMT) [11], or were terminally ill.

Ethical approval for this study was obtained from the Human Subjects Ethics Committee of The Hong Kong Polytechnic University. Permission to conduct the study was also sought from the person in charge of each center. Flyers introducing the aims of this study were posted at the centers. Older people who wanted to join the survey were enrolled through the centers. The eligibility of those wanting to participate in this study was assessed by a well-trained research assistant (RA) according to sample selection criteria. Written informed consent was obtained from each participant before they took part in a structured face-to-face interview. All of the interviews were carried out in private rooms in community centers to ensure confidentiality. On average, the interviews were completed within 45 min. All of the interviews were conducted by the RA according to a structured interview guideline developed by the principal investigator.

### Measurements

**Fig. 1:**
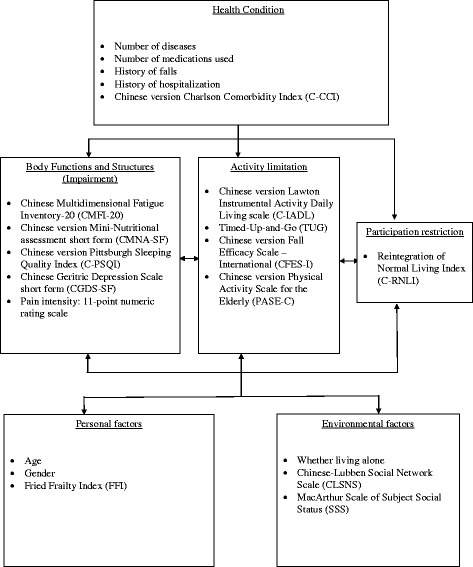
The measurements and demographic variables chosen in this study according to WHO-ICF

The WHO-ICF was used as the conceptual framework in this study to explore different risk factors associated with participation restriction among community-dwelling frail older people. All variables were systematically linked to the most appropriate ICF components [1] (Fig. 1).

### Dependent variables

**Fig. 2:**
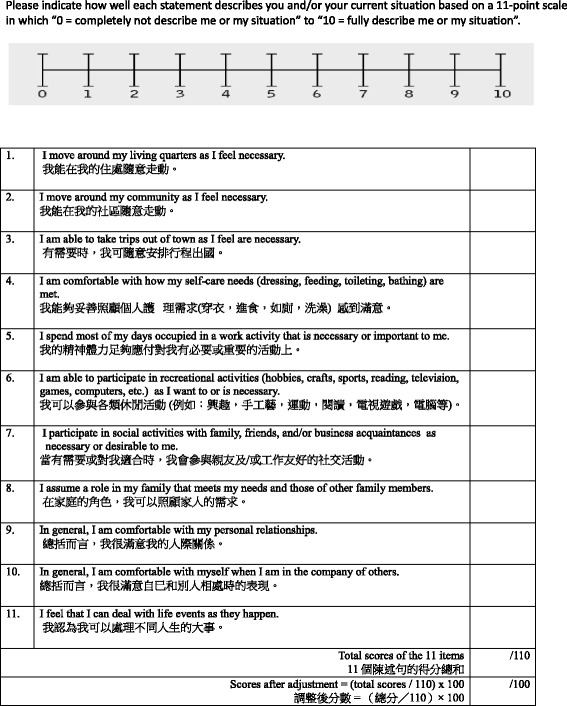
The Original Reintegration to Normal Living Index [12] and the Chinese-Reintegration to Normal Living Index. This was used to assess the level of participation restriction

Participation restriction is defined as how an older person’s health-related issues and personal and environmental hindrances limit that person’s involvement in valued life events [1]. The Chinese-Reintegration to Normal Living Index (C-RNLI), which was translated from the original RNLI [12], was used to assess the participation restriction of all participants. It consists of 11 declarative statements about different life events, such as involvement in social, civic, and recreational activities. Each item is rated on an 11-point numerical rating scale (with 0 indicating the least agreement and 10 the greatest agreement with the statements). Item scores were summed and proportionally converted to 100 through dividing the score by 1.1 to provide a total score; a lower score indicated a higher level of participation restriction. A confirmatory factor analysis conducted by the author (JL) showed that the C-RNLI is a two-factor structure scale (i.e., participation in physical activities and participation in social events) with an acceptable level of reliability and validity for measuring WHO-ICF participation restriction among community-dwelling frail older people. In the current study, the participants were identified as experiencing participation restriction if they scored 4 or below on any item in the C-RNLI. This criterion is similar to that used in Fairhall’s study [9] (Fig. 2).

### Independent variables

#### Personal and health factors

Are defined as the particular background of an individual’s life and health condition [1]*.* Demographic information on the participants, such as their age and gender, was collected. As mentioned in the section on sample selection criteria, the participants’ level of frailty was evaluated according to the occurrence of the five common characteristics of the frailty phonotype [10]. They were also asked to indicate the number of diseases, hospitalizations, and falls that they had suffered from in the past 12 months, and the prescribed medications that they were taking during that same period. The Chinese version of the Charlson Comorbidity Index (C-CCI) was used to assess levels of comorbidity among the participants. The C-CCI score is the sum of the comorbidity and age scores, with scores of 0, 1-2, 3-4, and *>* 5 representing a zero, low, medium, and high level of comorbidity, respectively [13].

#### Environmental factors

Have been defined as the physical, social, and attitudinal environment in which people live and conduct their lives [1]. Previous studies showed that environmental factors such as self-perceived socioeconomic status, living alone or with family, and social networks are associated with the development of frailty [14, 15]. These environment-related factors were explored in this study to determine whether they, too, are associated with participation restriction.

The social support network of the participants was assessed using the Chinese-Lubben Social Network Scale (CLSNS) [16, 17], which is a 10-item scale measuring five aspects of social networks: family network, friend networks, helping others, confidant relationships, and living arrangements. The total score ranges from 0 to 50. A higher score indicates a stronger social network. The participants’ self-perceived socioeconomic status was assessed using the MacArthur Scale of Subjective Social Status (SSS) [18]. The SSS denotes social status as a 10-rung ladder, with the top of the ladder representing people who are the best off and the bottom representing those who are the worst off. All of the participants were asked to mark an “X” on the rung that best represented their social status [18]. A higher step indicated a higher self-perceived social status. This scale has demonstrated high reliability and validity among various racial/ethnic groups and in various geographic locations [19]. The participants were also asked whom they were living with in the same household.

#### Body functions and structures (Impairment)

Have been defined as the bio-physio-psychological aspects of body systems or structures, and impairment as a significant loss of or deviation from body functions and structures. In terms of physiological functions, poor sleep quality and malnutrition are highly correlated with frailty [10, 20]. Fatigue is directly related to a decrease in muscle mass, which is one of the indicators of frailty [21]. Psychological issues such as depression also play a role in the development of frailty among the elderly, leading to participation restriction [22–25]. Measurements related to these areas have been included when exploring their association with participation restriction.

The participant’s level of fatigue was evaluated using the 20-item Chinese Multidimensional Fatigue Inventory-20 (CMFI-20) [26]. The total score, which ranged between 20 and 100, was obtained by summing up all item scores, with a higher score indicating a higher level of fatigue. The CMFI-20 was validated among 385 local cancer patients. A factor analysis revealed that the total score encompasses three factors (namely, physical, mental, and spiritual), with a factor loading ranging from 0.52 to 0.75. The Cronbach’s alpha for the three domains and the total score was between 0.7 and 0.8 [26]. This evidence supports the view that the CMFI-20 is a reliable and valid instrument.

Malnutrition is highly correlated with frailty [20]. Each participant’s level of nutrition was assessed using the Chinese version of the Mini-Nutritional Assessment – Short Form (CMNA-SF) [27], which is a tool widely used for nutritional screenings and assessments. The CMNA-SF has a maximum score of 14, with a score of 11 or below indicating that an individual is at risk of becoming malnourished or in a state of malnutrition. The CMNA-SF has been shown to be a good, validated tool for screening the nutritional status of older Chinese inpatients (sensitivity = 0.8955, specificity = 0.8800) and to be highly correlated (*r* = 0.838) with the full version of the MNA [28].

In this study, the Chinese version of the Pittsburgh Sleep Quality Index (C-PSQI) [29] was used to measure the participants’ subjective assessment of the quality of their sleep. The C-PSQI consists of 19 items grouped under seven domains, namely sleep duration, sleep latency, habitual sleep efficiency, sleep disturbances, subjective sleep quality, the use of sleep medications, and daytime dysfunction. The participants were asked to rate each item on a 0–3 scale. The total score ranges from 0 to 21, with higher scores representing poorer sleep quality. The C-PSQI is a reliable and valid tool for measuring sleep, with an overall reliability coefficient of 0.82 to 0.83, a sensitivity of 98%, and a specificity of 55% in screening insomnia among community-dwelling older people [30].

The presence of depressive mood among the participants was assessed using the Chinese Geriatric Depression Scale short form (CGDS-SF) [31]. The CGDS-SF consists of 15 items and each item is rated on a dichotomous scale. The total score, ranging from 0 to 15, is obtained by summing up all 15 items. A score of ≥ 6 indicates the presence of depressed mood. A Cronbach’s alpha of 0.89 indicating good reliability and a high criterion-related validity of 0.95 with a psychiatrist’s diagnosis of depression supports the view that the CGDS-SF is a reliable and valid tool for detecting the presence of depressive mood in older people [31].

The participants’ pain was measured using an 11-point numeric rating scale, as an association between pain and local frail older people was found in a previous cross-sectional survey [32].

#### Activity limitation

Has been defined as the difficulties that an individual faces in executing daily activities on a personal level. The Chinese version of the Lawton Instrumental Activities of Daily Living scale (C-IADL) was used to assess the participants’ independent living skills [33]. The C-IADL comprises nine skills of independent living, such as shopping, money management, meal preparation, and so forth, with each skill being scored on a scale of 0 to 3. The total score ranges from 0 to 27. A lower score indicates a greater level of dependence on the part of the participants. The C-IADL showed good reliabilities (i.e., test-retest reliability: 0.90; inter-rater reliability: 0.99). The construct validity was supported by the ability of the scale to differentiate between participants with different levels of functional performance in their daily activities [33].

The physical performance of individuals usually deteriorates as their frailty progresses to an advanced level, which can significantly affect their levels of activity [34]. In the current study, the Timed-Up-and-Go (TUG) test [35] was used to evaluate the participants’ mobility. In addition, it has been suggested that the fear of falling is one of the barriers to social participation among frail older people [23]. Thus, the Chinese version of the Fall Efficacy Scale – International (CFES-I) was used to measure the participant’s concern about falling when performing 16 different activities, from simple activities at home to more demanding outdoor activities [36, 37]. Each type of activity is rated on a 4-point scale, giving a summary score of 16–64, with higher scores indicative of more concern about falling. The CFES-I has been shown to have good reliability and construct validity across different studies [36, 38, 39].

The physical activity level of all of the participants was assessed using the 10-item Physical Activity Scale for the Elderly – Chinese (PASE-C) [40] to measure self-reported occupational, household, and leisure activities for the last week. Its total score is calculated by multiplying the amount of time spent in each activity (hours/week) by the weight of the pre-set item. A high score indicates a high level of physical activity. The PASE-C has been shown to be reliable and to have construct validity across different studies [41, 42].

### Data analysis

IBM SPSS Version 23.0 was used to run the statistical data analysis. Chi-square tests were used to compare proportions. The t tests of students were used to compare means in order to examine the associated risk factors of participants with or without participation restriction. A multiple logistic regression analysis was performed. Adjusted odds ratios (ORs) with corresponding 95% confidence intervals (CIs), and *p*-values were presented. ORs were used to evaluate risk factors associated or not associated with participation restriction. Demographic variables and variables related to health condition such as age, sex, number of diseases suffered as well as number of medications taken, and a history of falls were included in the model. The model also includes variables related to environmental factors, body functions and structures (impairment), and activity limitation. All statistical tests were two-tailed and variables were considered significant at a significance level of 0.05.

## Results

Two hundred and ninety-nine community-dwelling frail older people who were mainly female (*n* = 223, 74.6%) and whose mean age was 79.5 (SD 7.33) years were recruited. According to the FFI, 160 participants (53.5%) were identified as pre-frail, while 139 participants were classified as frail (46.5%). Among all of the participants, 207 (69.2%) were identified as having at least one participation restriction in at least one aspect of their life based on the C-RNLI. The top three events that participants reported of experiencing restriction mostly were “take trips out of town” (*n* = 170; 56.9%), “I assume a role in my family” (*N* = 115; 38.5%), and “I can deal with life events as they happen” (*N* = 97; 32.4%). On the other hand, “I am comfortable with how my self-care needs are met” had only 15 participants (5.0%) reported of having restriction. The mean C-RNLI was 68.3 (SD 19.43) among all of the participants in this study. Table 1 contains a summary of their characteristics according to whether or not they had experienced participation restrictions. Participants who were identified as having participation restriction were significantly older, frailer, weaker in the sense that they were suffering from more diseases and had a higher level of comorbidity, were of lower self-perceived socioeconomic status, and had weaker social networks, poorer body functions, and more activity limitations.

**Table 1:** Characteristics of the participants according to levels of participation restriction

	Total (*n* = 299)		With restriction (*n* = 207)		Without restriction (*n* = 92)		
	n	(%)	n	(%)	N	(%)	*P* value
Gender							0.298
Male	76	(25.4)	49	(23.7)	27	(29.3)	
Female	223	(74.6)	158	(76.3)	65	(70.7)	
Living Alone							0.859
Yes	118	(39.5)	81	(39.1)	37	(40.2)	
No	181	(60.5)	126	(60.9)	55	(59.8)	
Number of hospitalizations in the past 12 months							0.490
0	243	(81.3)	165	(79.7)	78	(84.8)	
1	45	(15.1)	33	(15.9)	12	(13.0)	
2	5	(1.7)	3	(1.4)	2	(2.2)	
3	5	(1.7)	5	(2.4)	0	(0.0)	
4	1	(0.3)	1	(0.5)	0	(0.0)	
Number of falls in the past 12 months							0.255
0	242	(80.9)	163	(78.7)	79	(85.9)	
1	41	(13.7)	29	(14.0)	12	(13.0)	
2	7	(2.3)	6	(2.9)	1	(1.1)	
3	8	(2.7)	8	(3.9)	0	(0.0)	
4	1	(0.3)	1	(0.5)	0	(0.0)	
	Total (*n* = 299)		With restriction		Without restriction		
	Mean	(SD)	Mean	(SD)	Mean	(SD)	*P* value
Personal Factors (Demographic Variables)							
Age	79.5	(7.33)	80.9	(7.04)	76.6	(7.12)	0.000**
Frailty phenotype criteria (0–5, 1–2: pre-frail; ≥3: frail)	2.54	(0.88)	2.72	(0.88)	2.11	(0.73)	0.000**
Health-related Factors							
Number of diseases	2.4	(1.49)	2.5	(1.52)	2.1	(1.38)	0.011*
Number of prescribed medications	3.1	(2.63)	3.2	(2.61)	2.7	(2.66)	0.130
C-CCI (0–43,a higher score means a higher level of comorbidity)	4.1	(1.13)	4.3	(1.08)	3.7	(1.12)	0.000**
Environmental Factors							
SSS (0–10, a lower rating means a lower self-perceived socioeconomic status)	4.5	(2.18)	4.3	(2.12)	5.1	(2.25)	0.006*
CLSNS (0–50, a higher score means stronger social networks)	22.5	(10.23)	20.9	(10.25)	26.2	(9.25)	0.000**
Body Functions and Structures (Impairment)							
Pain assessment (0–11, a higher rating means a higher level of pain)	3.9	(3.38)	4.2	(3.39)	3.1	(3.25)	0.013*
CMFI-20 (20–100, a higher score means a higher fatigue level)	66.5	(12.17)	69.4	(11.24)	60.1	(11.79)	0.000**
CMNA-SF (0–14, <11 indicates malnutrition)	12.6	(1.44)	12.4	(1.50)	13.1	(1.14)	0.000**
C-PSQI (0–21, a higher score means poorer sleep quality)	7.6	(4.10)	8.4	(4.10)	5.7	(3.46)	0.000**
CGDS-SF (0-15, ≥6 indicates the presence of depressed mood)	4.4	(3.58)	5.2	(3.61)	2.4	(2.60)	0.000**
Activity Limitations							
TUG (a longer time means a weaker physical performance)	18.4	(14.06)	21.4	(15.88)	11.8	(3.59)	0.000**
CFES-I (16–64, a higher score means more concern about falling)	33.3	(10.77)	35.8	(10.83)	27.4	(8.05)	0.000**
PASE-C (a higher score means a higher physical activity level)	65.7	(47.9)	54.9	(40.74)	90.7	(53.88)	0.000**
C-IADL (0–27, a lower score means a higher level of dependence)	20.4	(6.15)	19.0	(6.32)	23.5	(4.35)	0.000**
Participation Restriction							
C-RNLI (0–100, a lower score means a higher participation restriction level)	68.3	(19.64)	60.5	(18.41)	75.1	(18.08)	0.005**

*C-CCI* Chinese version Charlson Comorbidity Index, *SSS* MacArthur Scale of Subjective Social Status, *CLSNS* Chinese version of the Lubben Social Network Scale, *CMFI-20* Chinese version of the Multiple Fatigue Inventory-20, *C-MNA-SF* Chinese version of the Mini-nutritional assessment short form, *C-PSQI* Chinese version of the Pittsburgh Sleep Quality Index, *CGDS-SF* Chinese Geriatric Depression Scale short form, *TUG* Timed Up and Go Test, *CFES-I* Chinese version of the Fall Efficacy Scale-International, *PASE-C* Chinese version of the Physical Activity Scale for the Elderly, *C-IADL* Chinese version of the Lawton Instrumental Activities of Daily Living scale, *C-RNLI* Chinese Reintegration to Normal Living Index, *SD* Standard Deviation. *p < 0.05, **p < 0.005

Table 2 summarizes the results of the multiple logistic regression of participation restriction on all independent variables. A test of the full model with all independent variables against a constant-only model was statistically significant, with *χ*2 = 179.49, *p* < 0.001, indicating that the variables, as a set, reliably distinguished between those with or without participation restriction. The Nagelkerke R Square of the model was 0.671, and in 84.8% of cases the dependent variable (i.e., participation restriction) was correctly predicted by the model. Both collinearity measures [tolerance and the variance inflation factor (VIF)] were checked with regard to the impact of collinearity on the independent variables in the regression equation. The value of the tolerance ranged from 0.444 to 0.869 and the VIF was between 1.151 and 2.250 in the model, which indicated no small tolerance and a large VIF. In addition, no two independent variables were found with variance proportions greater than 0.50 under a conditioning index greater than 30. Therefore, no multicollinearity was evident.

**Table 2:** Logistic regression of participation restriction with other independent variables systematically related to the WHO-ICF framework

	Odds-Ratio (OR)	95% C.I.	
Personal Factors (Demographic Variables)		Lower	Upper
Age	1.05	0.97	1.14
Male	0.52	0.19	1.46
Frailty phenotype criteria (0–5, 1–2: pre-frail; ≥3: frail)	2.20*	1.10	4.42
Health-related Factors			
Number of diseases	1.22	0.87	1.73
Number of prescribed medications used	0.95	0.77	1.17
History of hospitalizations	0.99	0.42	2.37
History of falls	1.08	0.52	2.25
C-CCI (0–43, a higher score means a higher level of comorbidity)	1.14	0.67	1.96
Environmental Factors			
SSS (0–10, a lower rating means a lower self-perceived socioeconomic status)	0.79*	0.64	0.97
CLSNS (0–50, a higher score means stronger social networks)	1.00	0.96	1.05
Lives alone	0.71	0.28	1.81
Body Functions and Structures (Impairment)			
Pain assessment (0–11, a higher rating means a higher level of pain)	0.95	0.82	1.09
CMFI-20 (20–100, a higher score means a higher fatigue level)	1.00	0.96	1.04
CMNA-SF (0–14, <11 indicates malnutrition)	0.90	0.63	1.27
C-PSQI (0–21, a higher score means poorer sleep quality)	1.19*	1.05	1.35
CGDS-SF (0–15, ≥6 indicates the presence of depressed mood)	1.40**	1.15	1.70
Activity Limitation			
TUG (a longer time means a weaker physical performance)	1.21**	1.06	1.38
CFES-I (16–64, a higher score means more concern about falling)	1.05*	1.00	1.11
PASE-C (a higher score means a higher physical activity level)	0.99*	0.98	1.00
C-IADL (0–27, a lower score means a higher level of dependence)	0.92	0.82	1.04

*C-CCI* Chinese version of the Charlson Comorbidity Index, *SSS* MacArthur Scale of Subjective Social Status, *CLSNS* Chinese version of the Lubben Social Network Scale, *CMFI-20* Chinese version of the Multiple Fatigue Inventory-20; *C-MNA-SF* Chinese version of the Mini-nutritional assessment short form, *C-PSQI* Chinese version of the Pittsburgh Sleep Quality Index, *CGDS-SF* Chinese Geriatric Depression Scale short form, *TUG* Timed Up and Go Test, *CFES-I* Chinese version of the Fall Efficacy Scale-International, *PASE-C* Chinese version of the Physical Activity Scale for the Elderly, *C-IADL* Chinese version of the Lawton Instrumental Activities of Daily Living scale, *C-RNLI* Chinese Reintegration to Normal Living Index, *SD* Standard Deviation. *p < 0.05, **p < 0.005

The results show that the status of frailty, mobility evaluated by TUG, the fear of falling as measured by the CFES-I, sleep quality as measured by the C-PSQI, being depressed as measured by the CGDS-SF, subjective social status as measured by SSS, and physical activity level as measured by the PASE-C are significantly associated with participation restriction. The odds ratio of 2.20 (95% CI: 1.10–4.42) on the level of frailty implied that those who were frail and who had more frailty-related characteristics were twice as likely to experience participation restriction than those who were pre-frail. The odds ratio of 1.21 (95% CI: 1.06–1.38) on the TUG test implied that those who took a longer time to complete the TUG test were 1.21 times more likely to have participation restrictions than those could finish the test within a shorter time. Those who exhibited more depressed symptoms, had poorer sleep quality, and were more concerned about falling were more likely to have participation restrictions, with odds ratios of 1.40, 1.19, and 1.05, respectively. Those with lower self-perceived social status (SSS) and a lower physical activity level (PASE-C) tended to be more likely to have participation restrictions. The odds ratio of 0.99 (95% CI: 0.98–1.00) on PASE-C implied that the likelihood of participation restriction decreased by 1% [i.e., (1-0.99) x 100%] for each increase of one score in the PASE-C. The odds ratio of 0.79 (95% CI: 0.64–0.97) on SSS implied that the likelihood of participation restriction decreased by 21% for each increase of one score in social status.

## Discussion

Maintaining civil and social involvement by participating in different life events is important if older people are to keep up their satisfaction with life. However, information about the level of participation restriction among older people, particularly the pre-frail or frail, remains scant. The prevalence of participation restrictions among community-dwelling frail older people identified in the current study is about 70%, which is comparable with a previous study involving participants of a similar type [9] but obviously higher than in another study involving younger participants [43]. Many studies have in fact identified a positive correlation between age and participation restrictions [6, 9, 44].

When all factors conceptualized based on the WHO-ICF framework in running the multivariate model are included, 67% of the variance in participation restriction among the participants is explained. With the exception of factors under the health-related component, all components have at least one factor that is significantly associated with participation restriction. This finding supports the view that components in the WHO-ICF framework are interrelated and affect frail people’s levels of participation in daily, life, and social events. This provides further support for the argument that participation restriction is multifactorial in etiology [9]. The factors that show a significant association with participation restriction include the participants’ status of frailty, their self-perceived social status, level of exhibited depressive mood, sleep quality, mobility, level of fear of falling, and physical activity levels.

Among the different components of the WHO-ICF, “activity limitation” contains the largest number of risk factors significantly associated with participation restriction. Participants who took more time to complete the TUG test were more likely to have participation restrictions. Mobility in terms of the ability to move around in the nearby community was consistently identified in a previous study as a factor strongly associated with participation [6]. Beside mobility, balance confidence is another important factor in ensuring that older people are able to maintain their independence [45]. In this study, both the level of mobility and the fear of falling were significantly associated with participation restriction. This adds further support to the current evidence that for frail older people these are the two keys factors in maintaining a substantial degree of participation in different physical activities [4, 9]. Having lower levels of mobility and being overly concerned about falling will affect the physical activity levels of older people, and these three inter-related factors have been identified in this study as having a significant association with participation restriction.

Depressive mood, as reflected by the C-GDS, is the second-largest unique contributor to the variance in participation restriction in the current study. There is strong evidence of a relationship between depressive mood and restricted social participation, which is in agreement with previous findings that older people with depression have considerably higher odds of experiencing participation restriction [6, 9, 43]. The association between the quality of sleep of older people and their participation restriction has thus far seldom been explored. This study found that participants who reported having poorer sleep quality, as reflected by the C-PSQI, tended to have more participation restrictions. Poor sleep quality leads to tiredness during daytime, which may manifest as difficulty in sustaining a high level of functioning and reduced participation in different life events [46].

Frailty and disability were identified as two distinct but somewhat overlapping conditions commonly found in older people [8]. Frailty and disability coexist in about 67% of frail people [8]. Disability refers to a condition in which a person experiences substantial limitations in one or more major life activities, ranging from daily self-care activities (skills that are essential to living independently) to pursuits that are important to an individual’s life satisfaction [1]. In the WHO-ICF framework, participation restriction is regarded as an aspect of disability. Frailty has been identified as a personal risk factor that is significantly associated with participation restriction [9]. In this study, the participants’ status of frailty explains much of the variance in participation restriction.

Although a variety of factors were considered and all factors were included in running the regression model in this study, 33% of the variance in participation restriction was not explained by the multivariate model. In fact, each component in the WHO-ICF model is multi-dimensional in nature and contains many different factors. Those factors might not have been examined in this study. This may explain why no health-related factors were identified in this study as having a signification association with participation restriction.

The multifactorial nature of participation suggests that interventions should target the problems listed in the ICF framework in terms of functional levels. Fortunately, the majority of the risk factors identified in this study are modifiable. Thus, they can potentially be targeted in the effort to develop a multifactorial intervention to maintain or reduce participation restriction among frail older people. Depression, mobility, the fear of falling, and sleep quality are manageable in frail older people, and studies are warranted to investigate the productive values of all of these factors and the effect of targeting them during interventions aimed at enhancing the social participation of frail older people.

The findings should be interpreted with caution as this study has several limitations. First, levels of participation restriction in frail older people are a complicated construct in a complex population; they can never be completely captured by C-RNLI, which is a simple instrument. Some important elements under the concept of participation, such as learning and applying knowledge, may be restricted for different reasons that cannot be reflected in the current study. Second, another limitation in the current study is that there is limited empirical evidence to support the use of a cut-off value of 4 in any one the items of the C-RNLI as indicating the presence of participation restriction. Fortunately, a significant difference was identified in the majority of the data related to demographics, health, body functions, and activity levels, suggesting that the cut-off point of the C-RNLI used in this study can distinguish between two significantly different groups of older people with regard to their participation restriction. Third, due to a lack of instruments designed explicitly for the WHO-ICF conceptual framework, it is possible that factors have been misclassified under different components. Four, there is the potential limitation of sample selection bias. Participants were recruited from a district community or day care center where it can be presumed that participation in the survey involved some element of self-selection. This made it likely that some older people with participation restriction who never joined in any of the events / services offered by the district center would be excluded from this study. Also, older people with cognitive impairment were excluded from this study. All of these factors would limit the generalizability of its findings. Last, but not least, the cross-sectional design of this study prevented us from determining the casual relationship between participation restriction and the identified risk factors. Future studies are warranted to study the casual relationship between participation restriction and other risk factors that have been established in cross-sectional studies.

## Conclusion

Participation restriction is common among community-dwelling frail older people. It is associated with risk factors across different components in the WHO-ICF. This finding supports the view that participation restriction is multifactorial in nature. In light of the identification in this study of some modifiable risk factors, multifactorial interventions targeting those modifiable risk factors should be developed and evaluated in future studies so as to reduce participation restriction among frail older people.

## Abbreviations

C-CCI: Chinese version of the charlson comorbidity index
CFES-I: Chinese version of the fall efficacy scale-international
CGDS-SF: Chinese geriatric depression scale short form
C-IADL: Chinese version of the lawton instrumental activities of daily living scale
CIs: Confidence intervals
CLSNS: Chinese version of the Lubben social network scale
CMFI-20: Chinese version of the multiple fatigue inventory-20
C-MNA: Chinese version of the mini-nutritional assessment short form
C-PSQI: Chinese version of the Pittsburgh sleep quality index
C-RNLI: Chinese reintegration to normal living index
FFI: fried frailty index
ORs: Odds ratio
PASE-C: Chinese version of the physical activities scale for the elderly
SD: Standard deviation
SSS: MacArthur scale of subjective social status
TUG: Timed up and go test
